# Dynamic Fluctuations of Protein-Carbohydrate Interactions Promote Protein Aggregation

**DOI:** 10.1371/journal.pone.0008425

**Published:** 2009-12-23

**Authors:** Vladimir Voynov, Naresh Chennamsetty, Veysel Kayser, Bernhard Helk, Kurt Forrer, Heidi Zhang, Cornelius Fritsch, Holger Heine, Bernhardt L. Trout

**Affiliations:** 1 Department of Chemical Engineering, Massachusetts Institute of Technology, Cambridge, Massachusetts, United States of America; 2 Novartis Pharma AG, Basel, Switzerland; Dalhousie University, Canada

## Abstract

Protein-carbohydrate interactions are important for glycoprotein structure and function. Antibodies of the IgG class, with increasing significance as therapeutics, are glycosylated at a conserved site in the constant Fc region. We hypothesized that disruption of protein-carbohydrate interactions in the glycosylated domain of antibodies leads to the exposure of aggregation-prone motifs. Aggregation is one of the main problems in protein-based therapeutics because of immunogenicity concerns and decreased efficacy. To explore the significance of intramolecular interactions between aromatic amino acids and carbohydrates in the IgG glycosylated domain, we utilized computer simulations, fluorescence analysis, and site-directed mutagenesis. We find that the surface exposure of one aromatic amino acid increases due to dynamic fluctuations. Moreover, protein-carbohydrate interactions decrease upon stress, while protein-protein and carbohydrate-carbohydrate interactions increase. Substitution of the carbohydrate-interacting aromatic amino acids with non-aromatic residues leads to a significantly lower stability than wild type, and to compromised binding to Fc receptors. Our results support a mechanism for antibody aggregation via decreased protein-carbohydrate interactions, leading to the exposure of aggregation-prone regions, and to aggregation.

## Introduction

Protein glycosylation is a ubiquitous post-translational modification in cells. Many cell-surface and extracellular proteins are glycosylated, and play key structural and functional biological roles especially with respect to immunity and cell-cell recognition [1], [2], [3]. Protein glycosylation is also of substantial importance in Pharmacology. At least one third of approved protein therapeutics are glycoproteins [4]. Protein-carbohydrate interactions are involved in many molecular and cellular events such as cancer and lectin biology, cell adhesion and aggregation, and protein stability and function. For example, alternative protein glycosylation patterns are utilized or assessed as biomarkers of several cancer types [5], [6], where erroneous glycosylation perturbs native protein-carbohydrate interactions and cell adhesion. Differences in protein glycosyaltion of blood group antigens [7], [8], [9] also account for antibody-mediated agglutination of red blood cells when human ABO blood types are mismatched. Glycosylation of pharmaceutical erythropoietin has been engineered for improved *in vivo* stability and erythropoetic activity [10]. Glycosylation in the Fc region of IgG antibodies influences effector function via binding to Fc receptors, and antibody glycoforms with desired effector properties can be engineered [11], [12], [13]. Thus, protein glycosylation represents a structural variation of biological and pharmaceutical importance, especially with respect to protein stability, cell adhesion and aggregation.

While the discovery of protein-based and especially antibody therapeutics has seen a significant growth [14], [15], protein stability remains one of the most challenging tasks. Proteins undergo constant structural fluctuations leading to instability and loss of native monomer over time [16], [17]. Protein aggregation is the most common form of native monomer loss, and is deleterious not only because it may cause reduced therapeutic efficacy, but also because it may elicit an immune reaction in patients [18]. Protein aggregation is generally thought to occur via partially unfolded intermediates that with time and upon stress can form energetically-favorable aggregates. Understanding the aggregation mechanisms of therapeutic antibodies and other proteins would permit rational design of stabilization strategies.

**Figure 1:**
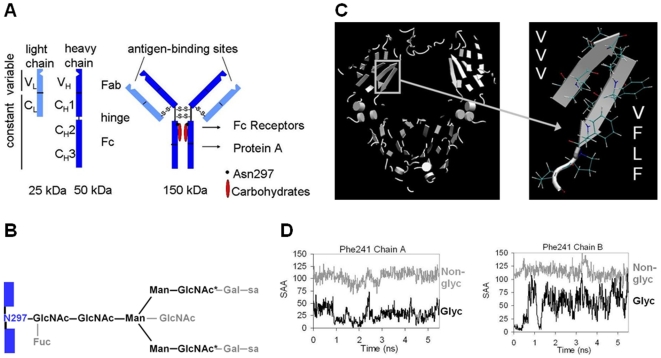
Computer simulations indicate dynamic fluctuations and increasing surface exposure of a hydrophobic carbohydrate-interacting residue. (A) a schematic of an IgG antibody. (B) N-linked carbohydrates at Asn297 that constitute the G0 glycosylation pattern are in black. The other heterogeneously included hexoses are in grey. (C) a view of all hydrophobic amino acids in the Fc region of IgG1 (polar and charged amino acids are excluded from this representation), and a close-up view of the most hydrophobic motif on the inner side of C_H_2 that interacts with carbohydrates. (D) SAA values calculated for Phe241 on chain A (left) and chain B (right) in the antibody Fc fragment assuming presence and absence of glycosylation.

Monoclonal antibodies (MAbs) are Y-shaped molecules comprising two Fab and one Fc domains, and are composed of two light and two heavy chains. The two heavy chains are further distinguished as chain A and chain B, and contain one variable domain, V_H_, and three constant domains for IgG, C_H_1, C_H_2, and C_H_3 (Figure 1A). Variable complementarity determining regions (CDRs) in the light and heavy chains of antibodies confer their tremendous diversity and hence the therapeutic opportunity to use biomarker-specific antibodies as pharmaceuticals. Of the different classes of antibodies, IgG, and more specifically isotype IgG1 and to a smaller extent IgG4 and IgG2, are most commonly used as biopharmaceuticals [19]. IgG MAbs are multidomain proteins with distinct melting transitions for Fab, C_H_2 and C_H_3 [20], [21]. In most human IgGs with stable Fabs, depending on sequences in the variable regions, C_H_2 is the domain with the lowest melting temperature. The human IgG C_H_2 domain is glycosylated at a single site, Asn297 (Figure 1A and 1B).

Carbohydrates can form a variety of non-covalent bonds with protein residues [22], and a ranking of sugar interface propensity for all amino acids has been proposed [23]. The hydroxyl groups of sugars make hydrogen bonds with amino acids such as Asp, Glu, Lys, Arg, His. At the same time, a hexose can have a conformation such that several of its carbon atoms are in a cluster that forms energetically favorable CH-pi interactions with parallel aligned aromatic rings, of for example Phe, Tyr or Trp [22], [24]. Two essential requirements listed for CH-pi interactions are that the sugar and aromatic rings are face to face and that they are less than 4Å apart. The N-glycosylation of an IgG antibody at a conserved residue, Asn297, exemplifies both of these types of interactions. Structural analysis of the antibody Fc region indicates a number of interactions between C_H_2 inner surface amino acids and neighboring carbohydrates [25], [26]. In particular, CH-pi interactions can form between sugars and the aromatic amino acids Tyr296, Phe241, Phe243 on each of the two chains in C_H_2. Crystal structures of human IgG-Fc show that Phe243 forms contact with GlcNAc [26].

There is a plethora of reports that compare different IgG glycovariants in stability and function. For example, gradual truncation of the carbohydrates at Asn297 in isolated Fc domains leads to a gradual decrease of the C_H_2 melting temperature (T_m_), with the least stable being the fully deglycosylated Fc [12]. A decrease in the melting temperature of the C_H_2 domain upon deglycosylation is observed for full antibodies as well [21]. Aglycosylated IgG also shows higher exposure of hydrophobic sites compared to native IgG [27]. IgG glycovariants that lack galactose (G0) have higher degree of mobility than their galactosylated (G1 and G2) counterparts, as evidenced by NMR analysis [28]. Moreover, increased surface exposure of GlcNAc in G0 glycovariants is associated with higher binding to mannose-binding protein (MBP) [29]. Another comparison indicates that the presence of terminal sialic acid in the Fc carbohydrates leads to increased anti-inflammatory properties of IgG [3].

At the same time, the aggregation mechanisms of natively glycosylated antibody domains remain unknown. The work presented here originated from the observation that the most hydrophobic motifs within Fc lie at the protein-carbohydrate interface in C_H_2, and that there are two aromatic amino acids in that region, Phe241 and Phe243. Thus, we hypothesized that disruption of sugar-protein, and more specifically CH-pi, interactions leads to exposure of aggregation-prone regions and to protein aggregation. We utilized a human IgG1 antibody with a single glycosylation site, and C_H_2 as the least stable domain, as a model system to investigate CH-pi sugar-protein interactions with respect to glycoprotein stability. There are different structural features of MAbs that contribute to aggregation, for example exposed hydrophobic regions [30], [31]. The antibody that we investigated as a model molecule has little exposed hydrophobicity within the CDRs and is fairly stable. Heat stress was used as the approach to protein destabilization in our accelerated aggregation experiments, with the assumption that our results would be applicable at lower, more physiological temperatures, albeit after a longer time. Successful connection between accelerated and long-term results for monoclonal antibodies is described in another manuscript from our group [32]. Here, our results from a variety of computational, fluorescence and molecular biology approaches indicate a reorganization of protein-carbohydrate interactions when the glycoprotein is stressed.

## Results

### Peptide-Carbohydrate Fluctuations Increase Surface Exposure of Phe241

Using the SAA (solvent accessible area) values from simulation, we tested whether a highly hydrophobic region within the Fc fragment that interacts with the carbohydrate moiety is dynamically exposed. A G0 glycosylation pattern (Figure 1B) for both chains A and B was used in the computer simulations. Two different SAA values were calculated: first, SAA with glycosylation; second, SAA without glycosylation (here the simulation was performed with glycosylation, but the sugar groups were removed only while calculating SAA). The SAA value with glycosylation indicates if the hydrophobic group is exposed to the solvent. Comparing this value with the SAA without glycosylation tells us whether this hydrophobic group is masked from the solvent through proximity to the Fc glycan. Figure 1 and Figure S1 show the SAA values for Phe241, Leu242 and Phe243 of the Fc fragment, each with two panels, one for heavy chain A, and one for heavy chain B. The side chains of Phe241 and Phe243 face the carbohydrates, while the side chain of Leu242 is buried in the protein domain fold. We observe that the SAA with glycosylation stays low without reaching the value of non-glycosylated case for Phe241 in chain A, and for Phe243 in chain A and chain B. Therefore, these residues are not significantly exposed to the solvent. The SAA values for Leu242 also remain low and do not depend on glycosylation. However, we notice that for Phe241 in chain B the SAA value for the glycosylated case increases and reaches the value of that of the non-glycosylated case. This shows that Phe241 gets exposed due to dynamic fluctuations of the carbohydrates, thereby leading to an exposed hydrophobic region that could cause aggregation.

### Protein-Carbohydrate Interactions Are Disrupted upon Heat Stress

**Figure 2:**
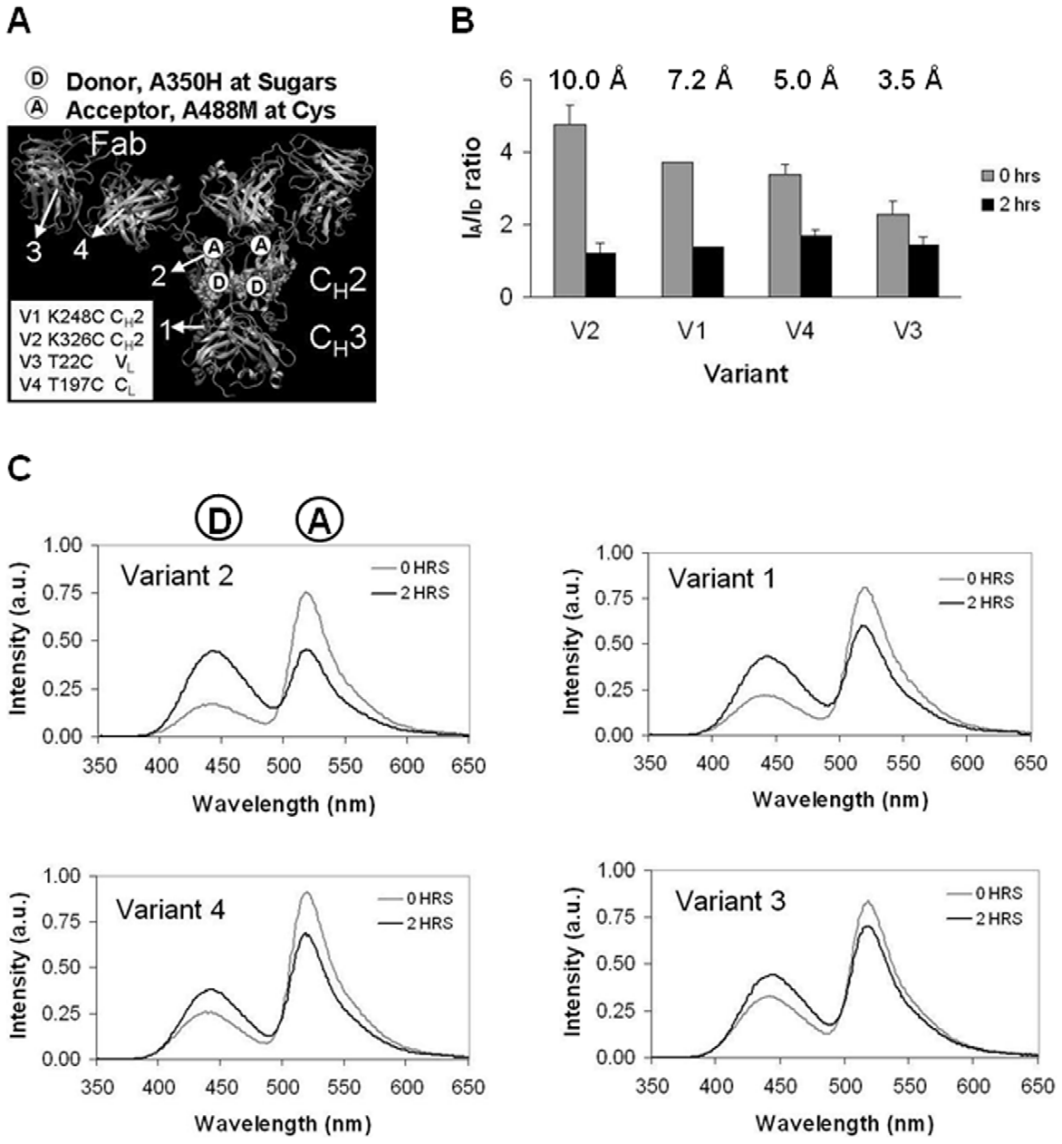
Peptide-carbohydrate interactions are disrupted upon heat stress. (A) a full-antibody cartoon illustrates the positions of the variants with engineered surface cysteines, and labeling approach in this set of experiments. Carbohydrates were labeled with Alexa350 hydrazide dye. Surface cysteines for each variant were labeled with Alexa488 maleimide dye. Variants 2 and 1 have surface cysteines engineered in the C_H_2 domain, and Variants 4 and 3 in the Fab region. (B) Ratiometric analysis of fluorescence data for double-labeled samples after heat stress at 60°C for 2 hours. Acceptor/donor ratios of stressed (2 hrs) and unstressed (0 hrs) samples are shown. The number above each pair of columns indicates the change in distance between the donor and acceptor fluorophores in the initial and final state. (C) Steady-state fluorescence data for individual double-labeled variants.

We monitored the dynamics of protein-carbohydrate interactions by fluorescence of labeled IgG1 antibody at specific sites. We generated 4 variants with a single surface-exposed amino acid substituted with cysteine (Figure 2A). Variant 1, K248C, carries a substitution at the C_H_2-C_H_3 junction, Variant 2, K326C, in C_H_2 at the lower hinge region, Variant 3, T22C, in V_L_, and Variant 4, T197C, in C_L_. Because of the presence of two chains in an antibody molecule, each variant has two surface cysteines, however in the figures presented here, the mutated sites on only one of the chains are shown. The cysteine variants, with glycosylation profile similar to wild type, were used for site-specific conjugation of maleimide-attached fluorophores (Figure 2 and Figure S2). On each variant, we also used hydrazide chemistry for attaching fluorophores at oxidized carbohydrates.

Variants 1–4 were expressed at comparable levels of 10–30 mg/L. The structures of all fluorophores used in this study are presented in Figure S2. The specificity of cysteine labeling was confirmed by proteolytic treatment and protein gel electrophoresis. Denaturing gel electrophoresis under reducing conditions permits distinction of labeling at the light or heavy chain for the four cysteine variants (Figure S2) and [33]. Peptide separation of endoprotease Glu-C and pronase-treated samples further points to the site-specific labeling of the variants (Figure S2). Each of the four variants, labeled at the engineered surface cysteines, remain more than 95% monomeric as determined by size-exclusion high performance liquid chromatography (SEC-HPLC) (Figure S2), and retain 80%–130% biological activity compared to wild type [33]. Specificity of labeling at the carbohydrates was confirmed by loss of fluorescence in the heavy chain upon PNGase F treatment; loss of monomer upon carbohydrate labeling up to 20% was observed by SEC-HPLC (Figure S2).

Each dual-labeled antibody variant was subjected to heat stress, and fluorescence emission spectra of stressed and unstressed samples were analyzed. The results indicate reduction of fluorescence resonance energy transfer (FRET) between donor and acceptor upon heat stress (Figure 2). For example, in the native state of Variant 2, there is a prominent peak for the acceptor fluorophore and a much smaller peak for the donor fluorophore, indicating high FRET and short distance between the fluorophores. When Variant 2 is stressed, the acceptor peak decreases and the donor peak increases, indicating less FRET and longer distance between the fluorophores in this condition. The distance between the donor and acceptor fluorophores at the final state increases the most for Variant 2 (10.0 Å), and to a smaller extent for Variant 1 (7.2 Å), Variant 4 (5.0 Å) and Variant 3 (3.5 Å). The average of donor to acceptor intensity ratios and the calculated distances in the native (time 0 hrs) and stressed (time 2 hrs) states are shown in Figure S2.

### Protein-Protein and Carbohydrate-Carbohydrate Interactions in the C_H_2 Domain Increase upon Stress

**Figure 3:**
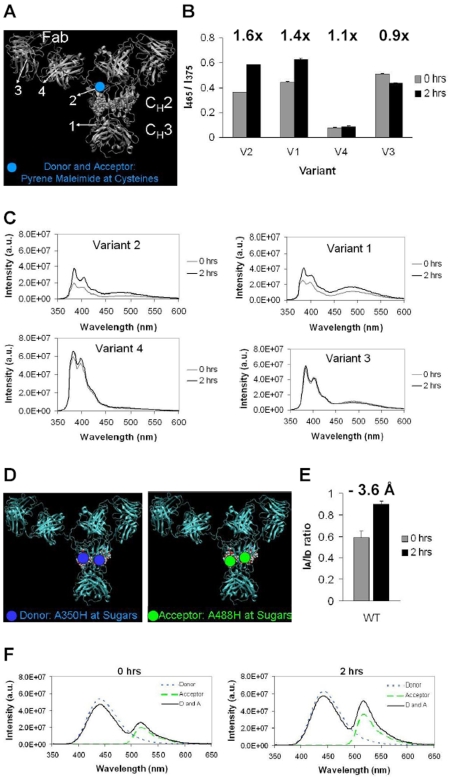
Protein-protein and carbohydrate-carbohydrate interactions in the C_H_2 domain increase upon stress. (A) Protein intermolecular interactions via pyrene-labeled cysteine variants. (B) Excimer ratio of non-stressed (0 hrs) and stressed (2 hrs at 60°C) samples. The numbers above the pair of columns for each variant indicate the ratio of stressed (value of column in black) to unstressed (value of column in grey) sample ratios. (C) Emission spectra of pyrene-labeled variants. (D) Experimental set-up for analyzing carbohydrate intermolecular interactions. Antibody samples with carbohydrates labeled with donor, A350, or acceptor, A480, were analyzed separately and as a mixture. (E) Ratios of acceptor and donor intensity in the mixed samples before and after stress at 60°C for 2 hours. The change in intermolecular average distance between the fluorophores after stress is shown above the columns. (F) Fluorescence spectra of Donor only, Acceptor only, and Donor and Acceptor mixture for each condition.

We used the same set of cysteine variants to monitor protein-protein interactions. This time, pyrene maleimide was used for conjugation to the engineered surface cysteines in Variants 1–4. Pyrene is a fluorescent molecule with a characteristic increase in emission at 465 nm, referred to as excimer, when two pyrene molecules are in immediate (<10Å) proximity. Variants 1 and 2 show an increase by 140% and 160% in excimer fluorescence upon stress at 60°C for 2 hrs; Variants 3 and 4 show a very slight decrease or increase of excimer fluorescence (Figure 3A–3C). Thus, protein-protein interactions increase specifically in the C_H_2 domain upon heat stress.

In addition, we probed for carbohydrate-carbohydrate intermolecular interactions by FRET. One population of antibody wild type was labeled at the carbohydrates with donor (Alexa350 hydrazide), and another with acceptor (Alexa488 hydrazide). Donor, acceptor and donor-and-acceptor samples were analyzed before and after stress (Figure 3D–3F). After heating at 60°C for 2 hours, the mixture of donor and acceptor produces more FRET (higher I_A_/I_D_ ratio) than the non-stressed sample, indicative of increased intermolecular interactions of the carbohydrate moieties. The calculated distances from the FRET results correspond to carbohydrates of neighboring antibody molecules being closer by 3.6 Å on average after stress.

### Carbohydrate Interactions with Aromatic Amino Acids Are Important for C_H_2 Stability

**Figure 4:**
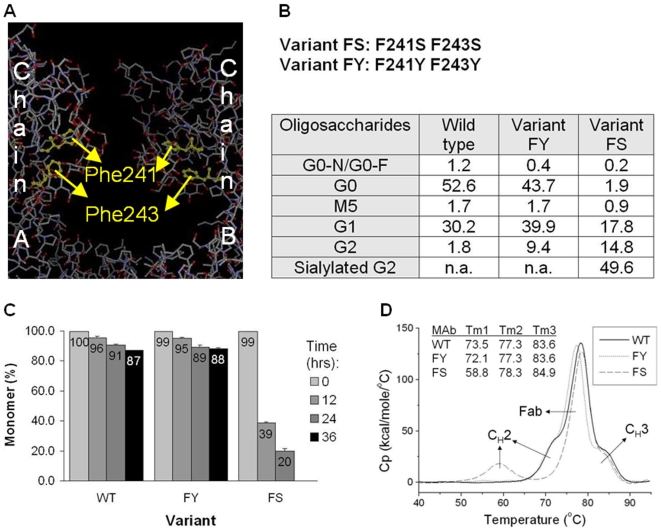
The IgG1 variants F241S F243S (FS) and F241Y F243Y (FY) illustrate the importance of CH-pi interactions for stability of the C_H_2 antibody domain. (A) Close-up on the C_H_2 domain showing the location of the mutated residues on the inner side of C_H_2, and the alignment of Phe241 and Phe243 aromatic rings with sugar rings. (B) Glycosylation profile of wild type and the two F241 F243 variants. The percentage of the predominant glycoforms is listed. (C) Accelerated aggregation of wild type and variants at 58°C for the indicated time. Percent monomer at each time point was calculated from SEC-HPLC analysis. (D) DSC thermograms of wild type and Variants FY and FS.

To further explore the role of protein-carbohydrate interactions experimentally, we generated two antibody mutants, F241S F243S (Variant FS), and F241Y F243Y (Variant FY) (Figure 4A). Variant FS has Phe residues, known to interact with the carbohydrate moiety [25], [26], replaced with polar serine residues that have smaller and non-aromatic side chains. In Variant FY the same Phe residues are replaced by Tyr residues, suggested to have higher sugar interface propensity [23]. At the same time, other hydrophobic residues, for example Val264, remain unmodified in this region. Both wild type and Variant FY have very little if any sialylation of their carbohydrates, while nearly 50% of the molecules of Variant FS bear at least one sialic acid residue (Figure 4B).

The stability of wild type and Variants FS and FY was compared in accelerated aggregation experiments and by differential scanning micro-calorimetry (DSC). Samples at 150 mg/ml were induced to aggregate at 58°C for up to 36 hrs, and monomer levels were resolved and quantified by SEC-HPLC (Figure 4C). Wild-type monomer levels gradually decrease from 100 to 96, 91, and 87% for 0, 12, 24, and 36 hrs time points. Variant FY trails by 1–2% in the earlier time points but is at 88% at 36 hrs, within statistical error of wild type. Variant FS is significantly less stable at this temperature showing a monomer decrease from 99% to 39% at 12 hrs, and to 20% at 24 hrs. The 36-hrs samples were not run on SEC-HPLC because of the presence of abundant visible aggregates.

DSC results also differentiate between the variants and wild type (Figure 4D). The melting temperature (T_m_) of the C_H_2 domain decreases from 73°C for wild type to 59°C for Variant FS. Minor differences in Variant FS, not greater than 1°C, are observed for the melting transitions of Fab and C_H_3 as well. Although the C_H_2 melting transition shoulder of Variant FY overlaps that of wild type, the software fitting indicates lowering of the C_H_2 Tm to 71°C, while the other two Tm's remain unchanged.

We carried out a number of additional experiments to compare Variant FS to wild type, and to better understand the observed decrease in stability. Variant FS retains the β-sheet-rich structure as wild type (Figure S3A). The variant has different mobility patterns compared to wild type on reducing as well as native gel electrophoresis (Figure S3B and S3C). We also carried out protease treatment experiments to compare protein surface exposure in Variant FS and wild type. Digestion of the antibodies with Glu-C is more efficient (more small fragments) for Variant FS than wild type; that efficiency is largely equalized in the variant and wild type deglycosylated counterparts, although some differences persist (Figure S3D). Furthermore, Variant FS retains full FcRn and partial FcγRIa binding function but loses binding to FcγRII and FcγRIII receptors (Table 1 and Table 2).

**Table 1:** Equilibrium dissociation constants K_D_ of antibody wild type and Variant FS to Fc receptors.

Mab	FcγRIIa	FcγRIIb	FcγRIIIa F158	FcγRIIIa V158	FcγRIIIb	FcRn pH 6.0
WT	5.4 µM	24 µM	12 µM	5.0 µM	30 µM	2.6 µM
FS	No binding	No binding	No binding	No binding	No binding	2.7 µM

**Table 2:** Kinetic rate constants k_on_ and k_off_ of antibody wild type and Variant FS binding to FcγRIa.

MAb	k_on_ (M^−1^s^−1^)	k_off_ (s^−1^)	K_D_ (M)	R_max_ (RU)
WT	4.5×10^4^	8.2×10^−4^	1.8×10^−8^	479
FS	2.9×10^4^	2.1×10^−3^	7.4×10^−8^	126

## Discussion

Protein-carbohydrate interactions present a high level of diversity and complexity in Biology and Pharmacology. We used an antibody with a single glycosylation site to monitor the dynamics of protein-carbohydrate interactions. Our results point to intramolecular dissociation of carbohydrates from the adjacent protein surface, and to increased intermolecular protein-protein and carbohydrate-carbohydrate interactions upon stress. These dynamic fluctuations promote antibody aggregation.

Our interest in protein-carbohydrate interactions with respect to antibody stability originated from the observation that the most hydrophobic motif in Fc is on the inner surface of C_H_2 that interacts with carbohydrates. Moreover, computer simulations on Fc of human IgG1 antibody provided initial evidence of the transient exposure of hydrophobic amino acids in the C_H_2 domain. Even for as short of a time as 5 ns, one of the hydrophobic carbohydrate-interacting residues, Phe241 on chain B, gets nearly as exposed as the that residue would be in a non-glycosylated Fc (Figure 1D). The results for Leu242 represent a useful negative control since this amino acid is oriented inward towards the immunoglobulin fold, and low surface exposure is not affected by the presence or absence of carbohydrates (Figure S1). We do not observe change in SAA values for Phe243 in Fc simulations (Figure S1), and for Phe241 and Phe243 in full antibody simulations (not shown). It is possible that the full antibody provides an additional rigidity to the Fc domain, and thus only simulations on the more flexible Fc domain pick up differences from thermodynamic fluctuations. Still, the presented data provides valuable and unprecedented information for a very computationally demanding system. The positive result for Phe241 encouraged us to explore the protein-carbohydrate interactions with respect to protein stability in further detail.

Combinations of fluorophores attached at specific sites on proteins permit intermolecular and intra-molecular FRET experiments that reveal increase or decrease of interactions at different conditions. We utilized a pair of fluorophores, Alexa350 and Alexa488 that can undergo energy transfer when the fluorophores are close together, with R_0_ of about 50 Å, the Förster distance at which energy transfer is 50% efficient (Invitrogen). For comparison, the length of the Fab and Fc IgG domains is about 50 Å each, with the full antibody length varying between 50 Å and 100 Å, depending on the extension of the Fab domains from Fc in the hinge region. We also utilized another fluorophore, pyrene, with a characteristic excimer peak when two pyrene molecules are less then 10 Å apart. Our intramolecular FRET experiments with Alexa350 and Alexa488 indicate less FRET between the donor-labeled carbohydrates and acceptor-labeled adjacent protein residues when the samples are stressed than in the original samples. This result is most pronounced for variant 2 that has a labeled surface cysteine in the lower hinge region of C_H_2. Consistent with the further location of the labeled cysteines in Variants 1, 4, and 3 from the carbohydrate moiety, a smaller difference in FRET is observed for these variants upon stress than for Variant 2.

Although we used accelerated stress conditions of high protein concentration and high temperature for this set of FRET experiments, we expect that our results will be representative for lower temperature conditions after a longer stress time. We established a mathematical model that successfully predicts long-term aggregation data based on accelerated aggregation experiments [32]. The fluorophores we used in our experiments as site-specific reporters of structural dynamics are extrinsically attached probes with properties on their own. However, the fact that labeled antibodies retain most of their activity indicates that the fluorophores do not perturb the structure of the antibodies. With the consistent comparison of fluorescence across all four variants, we believe our results represent the variants' site dynamics and not the probes' intrinsic properties. Moreover, when we label K326C (Variant 2) in the antibody wild type and in the Variant FS background, we observe two times more aggregates in Variant K326C FS than in Variant K326C WT (not shown), consistent with the lower stability of Variant FS compared to WT. This result suggests that the aggregation patterns we observe of our labeled samples are a function of the unlabeled protein stability, and not of the attached fluorophore.

Many mutations have been generated in the Fc region mostly for the purpose of modulating Fc Receptor binding [34]. Thus, there is abundant literature on amino acid mutations known to affect receptor binding or glycosylation pattern [34], [35]. However, few C_H_2 mutations have been used in stability analysis. Variant FS (F241S F243S) and FY (F241Y F243Y) permit elucidation of the role of protein-carbohydrate interactions in C_H_2 domain stability. Unlike other destabilized C_H_2 antibody variants such as deglycosylated variants [12], Variant FS is more fully glycosylated (Figure 4B). Similarly to the finding that deglycosylated human IgG shows higher protein hydrophobicity exposure [27], Variant FS is more susceptible to proteolytic digest (Figure S3D), indicative of higher exposure of protein surface than wild type. The different Glu-C digestive pattern of deglycosylated wild type and Variant FS suggests the presence of structural differences apart from carbohydrate-protein binding. Structural differences between Variant FS and wild type are also reflected in the loss of binding of the variant to FcγRII and FcγRIII receptors. Altogether, the results indicate that in Variant FS the carbohydrates and protein form fewer contacts, and that disruption of carbohydrate-pi interactions leads to structural changes and to a significant reduction in antibody stability.

In contrast to Variant FS, Variant FY (F241Y F243Y) carries a conservative mutation with respect to CH-pi interactions, and remains fairly stable. Variant FY trails wild type by only 1–2% in accelerated aggregation experiments at 12 and 24 hrs, and narrowly surpasses wild type at 36 hrs. We do not believe that the observed differences in aggregation kinetics are a result of the higher G2 glycosylation levels of Variant FY because another wild type sample (Figure S2) has similar G2 glycosylation levels. The change of aggregation kinetics in Variant FY in comparison to wild type may occur if the introduced mutation stabilizes a subpopulation of the original sample or an intermediate.

**Figure 5:**
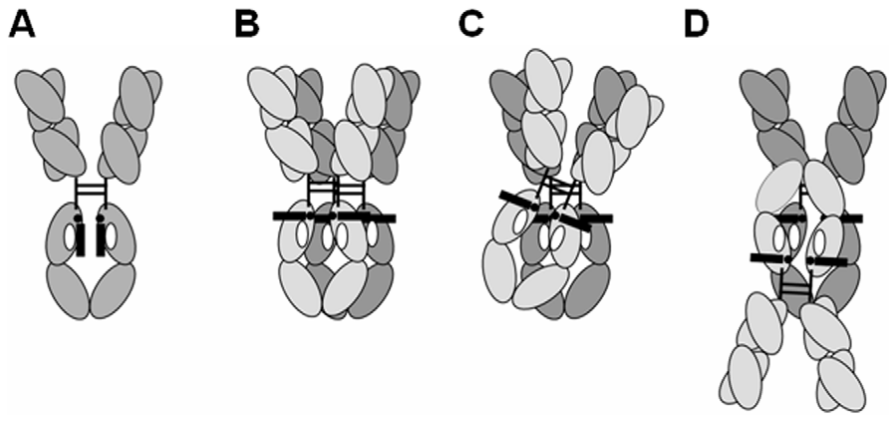
Model of an aggregation mechanism of IgG antibodies. (A) Native antibodies have carbohydrates that interact with neighboring amino acids on the inner side of C_H_2. The light and heavy chains are presented in grey. The sugars are shown as a black bar, attached to Asn297, a black dot. White ovals represent a patch in C_H_2 that is usually masked by the carbohydrates. (B–D) Dynamic motion of carbohydrates transiently exposes underlying protein hydrophobic residues. Stress, such as heat, exacerbates that motion, exposing aggregation-prone region, and resulting in protein aggregation where new glycoprotein interactions are formed. One antibody molecule is in light grey, and the other in dark grey. (B) Parallel stacking of full antibodies resulting from interactions between newly exposed protein patches. (C) Angled stacking for improved interaction between exposed protein patches. (D) anti-parallel stacking of full antibodies with interactions between newly exposed protein patches.

Antibody molecules can assume a number of orientations during aggregation. Our results point to interactions in the C_H_2 domain as a mechanism of antibody aggregation. Such interactions may occur by parallel, angled or anti-parallel stacking of molecules (Figure 5). We suggest the angled orientation as an alternative to the parallel stacking for improved 3-dimensional distribution of the Fab domains. A salient feature in all three examples is the presence of intermolecular interactions between protein patches that become exposed due to protein-carbohydrate fluctuations. For simplicity, here we discuss models of interactions between the dynamically exposed patches, although in reality such patches may interact also with existing surface-exposed patches.

In the parallel and angled orientations, the molecules are only partially overlapping, so that the front patch of one molecule can interact with the back patch of the other molecule; in contrast, the anti-parallel orientation has both Chain A and Chain B of the molecules overlap to facilitate proximity of the patches from one of the chains. The fluorescence results presented in this report suggest increased protein interactions in the C_H_2 domain but no change in the light chain in Fab, and at the same time increased proximity of carbohydrates from adjacent molecules. The Fab domains are the furthest apart in the anti-parallel orientation, but at the same time the carbohydrates, the lower hinge region of C_H_2, and the C_H_2-C_H_3 junction, are the closest in the parallel or angled orientation. Although all three orientations are possible, as well as others, additional fluorescence experiments can delineate the predominant aggregating orientation.

Collectively, our results point to a mechanism of antibody aggregation where native protein-carbohydrate interactions are disrupted, and newly exposed sites interact and contribute to aggregation. This model is consistent with previously reported results. It has been shown that partially or fully deglycosylated C_H_2 domains have lower melting transitions than wild type (a mixture of G0, G1, G2, and sialylated antibody) [12]. In addition, removal of carbohydrates leads to a closed conformation of C_H_2 [26], suggestive of the capacity of the usually protected protein surfaces to interact. Furthermore, dynamic motion of G0-glycosylated antibodies has been observed by NMR, and increased carbohydrate exposure has been implicated in activating the complement system via interactions with the mannose-binding lectin (MBL) [28], [29].

The molecular level insights from our work extend to at least two areas. First, the correlation of our results and the literature on MBL binding suggests that antibody aggregation may be immunogenic not only because antibody multimers are perceived as foreign, but also because stress- and time-induced exposure of antibody carbohydrates leads to activation of the complement system via MBL. Second, our results suggest an aggregation mechanism for antibodies generated against carbohydrate-based targets. Considering the prevalence of aromatic, hydrophobic residues in carbohydrate binding sites, most antibodies that come out of the Discovery phase in pharmaceutical research to target carbohydrates are likely to have Phe, Tyr or Trp in their CDR regions. The high surface exposure of such amino acids is likely to be a driving force of intermolecular interactions that lead to high aggregation propensity *in vitro*. Thus, the results presented here indicate the importance of antibody protein-carbohydrate dynamic interactions in stability and function.

## Materials and Methods

### Computer Simulation

Molecular dynamics simulations were performed for the Fc fragment of a human IgG1 antibody using the CHARMM simulation package [36]. Here we used an explicit solvent model. The CHARMM fully atomistic force field [37] was used for the protein, and TIP3P [38] model for water. The simulations were performed at 300K and 1atm in the NPT ensemble. The Fc fragment was simulated with the inclusion of disulphide bonds in the hinge region. A G0 glycosylation pattern was used where the parameters for the sugar groups were derived in consistence with the CHARMM force field, following from the CSFF force field [39].

### DNA Vectors and Mutagenesis

Vectors encoding the light and heavy chains of a proprietary human IgG1 antibody were kindly provided by Dr. Burkhard Wilms at Novartis. The light and heavy chain genes were subcloned in vector gWIZ (Genlantis), engineered for protein expression by transient transfection of mammalian cells. Antibody variants were either *de novo* synthesized (GeneArt) or generated by in-house site-directed mutagenic PCR and confirmed by sequencing.

### Protein Expression and Purification

Antibody wild type and variants were expressed at 10–100 mg levels by transient transfection of Freestyle HEK 293 cells (Invitrogen) with polyethyleneimine (Polysciences) as the transfection reagent. Cell culture supernatant was collected 7–10 days post-transfection. Antibodies were purified on a protein A column (GE Healthcare), eluted with 50 mM citrate buffer, pH 3.5, and buffer exchange in 20 mM His, pH 6.5 buffer for stability analysis or 50 mM Tris buffer for fluorescence labeling.

### Protein Labeling

Labeling of cysteine variants was carried out first by treatment with L-cysteine to decap the engineered surface cysteine, followed by buffer exchange into 50 mM Tris/EDTA, and incubation with 5–10-fold excess of Alexa488 maleimide dye (Invitrogen) for 1 hr at room temperature or with 10-fold excess of Pyrene maleimide dye (Invitrogen) for 12 hrs at room temperature. After removal of free dye, and buffer exchange to 50 mM phosphate buffer pH 7.0, the efficiency of protein labeling was calculated as mole of dye per mole of protein according to manufacturer's protocols (Invitrogen). Labeling with Alexa488 yielded efficiency of 1.5–2.0, and with Pyrene of 0.8–1.0.

Antibody carbohydrates were labeled with Alexa350 hydrazide and Alexa488 hydrazide (Invitrogen) following a carbohydrate labeling protocol (Pierce), using sodium *meta*-periodate for oxidation of carbohydrate hydroxyl groups to aldehydes, and protein incubation with 10-fold dye excess at room temperature for 12 hrs. Labeling efficiencies were in the 1.0–1.5 range of mole dye per mole protein.

### Fluorescence Analysis

Steady-state fluorescence experiments were carried out on Fluorolog (Horiba Jobin Yvon). Samples at 50 mg/ml were stressed at 60°C for 2 hours, diluted 30-fold and placed on ice until analysis. For pyrene analysis, excitation was at 342 nm, and emission was recorded from 347 to 600 nm. The ratio of excimer signal intensity at 465 nm to a basal signal intensity at 375 nm was calculated for each samples. Samples labeled with Alexa350 and Alexa488 were excited at 347 nm, and emission was recorded from 350 to 650 nm. Distances between Alexa fluorophores were calculated assuming Förster's distance R_0_ for Alexa350 and Alexa488 of 50Å and κ^2^ of 2/3. Then, change in distance upon stress from time t = 0 hrs (r_0_), to time t = 2 hrs (r_2_) was calculated based on the measured emission intensity of donor and acceptor (I_D_ and I_A_) at the corresponding time points with the equation:
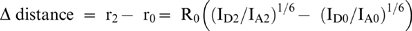



### Stability Analysis

Size-exclusion High Performance Liquid Chromatography (SEC-HPLC) was used to determine monomer loss over time in accelerated aggregation experiments. Antibody wild type and variants FS and FY were incubated at 150 mg/ml at 58°C for up to 36 hours. For each time point, sample aliquots of 2 µl were diluted 15-fold in 15 mM potassium phosphate buffer, pH 6.5 to 10 mg/ml. Monomers were resolved from non-monomeric species by SEC-HPLC on a TSKgel Super SW3000 column (TOSOH Bioscience), kept at 22°C, with mobile phase 150 mM potassium phosphate, pH 6.5, and flow rate of 0.2 ml/min. Percent monomer was calculated as the area of the monomeric peak divided by the total area of all peaks detected at 280 nm.

The thermodynamic stability of antibody wild type and variants FS and FY was compared by Differential Scanning Micro-calorimetry (DSC, Microcal). Samples were analyzed at a concentration of 2 mg/ml in 20 mM His pH 6.5 buffer and at 1.0°C/min scan rate. The sample data was analyzed by subtraction of the reference data, normalization to the protein concentration and DSC cell volume, and interpolation of a cubic baseline. The peaks were deconvoluted by non-2-state fit using Microcal Origin 5.0 software.

PNGase F (New England Biolabs) and Glu C (Sigma) were used for protein digests according to manufacturers' protocols. Reduced and non-reduced antibody samples were resolved on SDS-PAGE (BioRad). Native gel electrophoresis was carried out in 1% agarose gels in 30 mM His, pH 6.0 buffer system.

### Glycosylation Pattern Analysis

Glycans of the MAb wild type and variants were released with PNGase F. After separation of the oligosaccharides from the protein, the glycans were labeled with 2-Amino-benzamide. The excess dye was removed prior to normal phase liquid chromatography (NP-LC) measurements, performed with an Agilent 1100 HPLC coupled to Bruker Esquire 3000 ion trap mass spectrometer. Glycan identification was performed by referencing NP-LC retention times and comparing measured with theoretical molecular weight. Percent peak area was obtained by integrating the fluorescence-label signals and comparing with the total peak area.

### FcR Binding

Recombinant extracellular domains of Fc receptors were immobilized covalently or through enzymatic, site-directed biotinylation on suitable sensorchips, and affinity of antibodies to Fc receptors was determined through steady-state analysis at pH 7.4 (FcγRII, FcγRIII) or pH 6.0 (FcRn) or kinetic analysis at pH 7.4 (FcγRIa) on a Biacore 3000 instrument.

## Supporting Information

Figure S1 Computer simulations
(0.11 MB DOC)

Figure S2 Results from FRET experiments
(0.23 MB DOC)

Figure S3 Structural comparison of wild type and Variant FS
(0.77 MB DOC)
