# Supplementary material for: Dynamic Fluctuations of Protein-Carbohydrate Interactions Promote Protein Aggregation
Source: PLoS One. 2009 Dec 23;4(12):e8425. doi: 10.1371/journal.pone.0008425 (PMC2791859; doi:10.1371/journal.pone.0008425)
Supplement: Figure S1 — Computer simulations (0.11 MB DOC) [file pone.0008425.s001.doc]

**Figure S1**

**A**

**
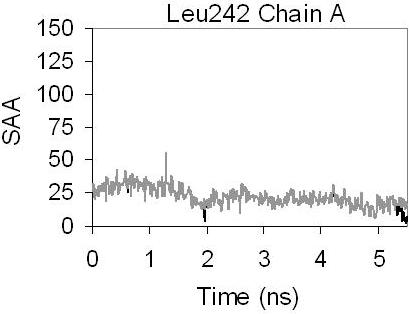

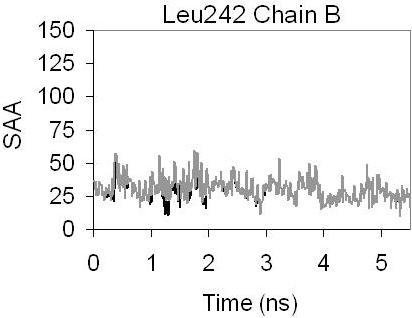
**

**Glyc**

**Non-**

**glyc**

**Glyc**

**Non-**

**Glyc**

**B**


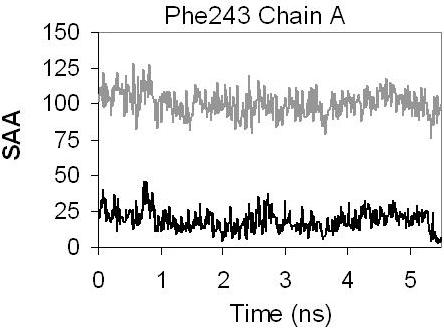

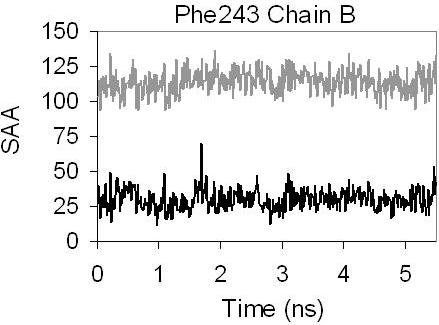


**Non-**

**glyc**

**Glyc**

**Glyc**

**Non-**

**Glyc**

**Figure S1. Computer simulations.**

(A) SAA values calculated for Leu242 on chain A (left) and chain B (right). (B) SAA values calculated for Phe243 on chain A (left) and chain B (right).
