# Supplementary material for: Dynamic Fluctuations of Protein-Carbohydrate Interactions Promote Protein Aggregation
Source: PLoS One. 2009 Dec 23;4(12):e8425. doi: 10.1371/journal.pone.0008425 (PMC2791859; doi:10.1371/journal.pone.0008425)
Supplement: Figure S2 — Results from FRET experiments (0.23 MB DOC) [file pone.0008425.s002.doc]

**Figure S2**

**A B**


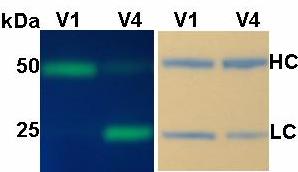

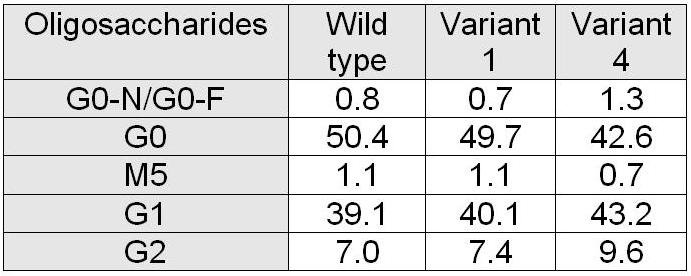


**C D**

**
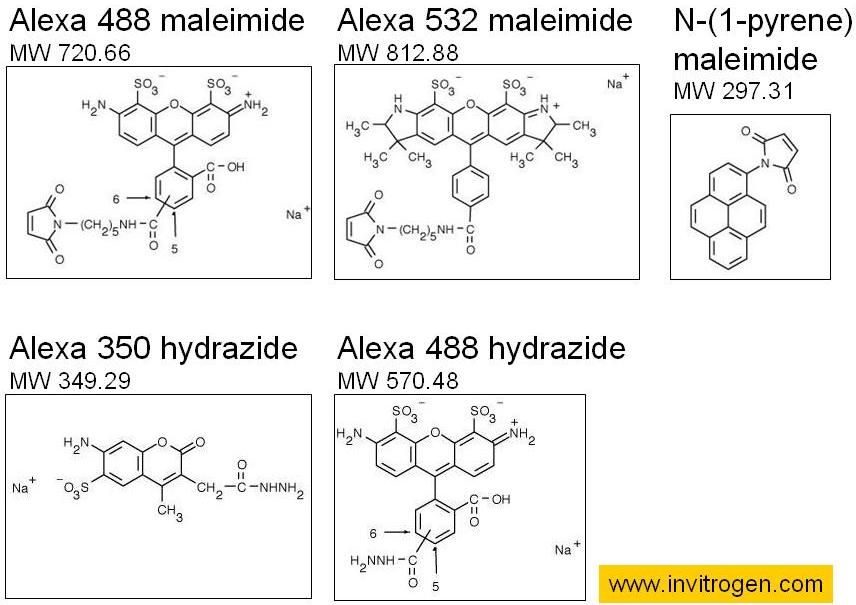

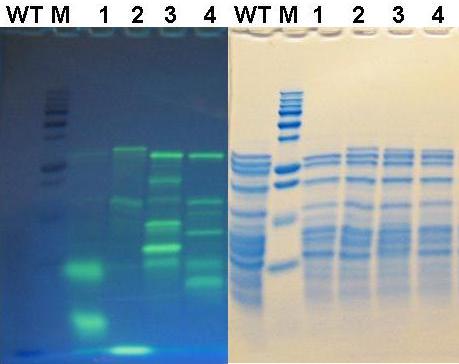
**

**E F**

**
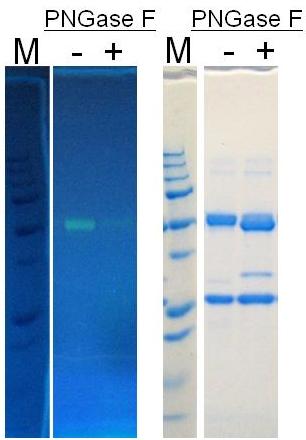

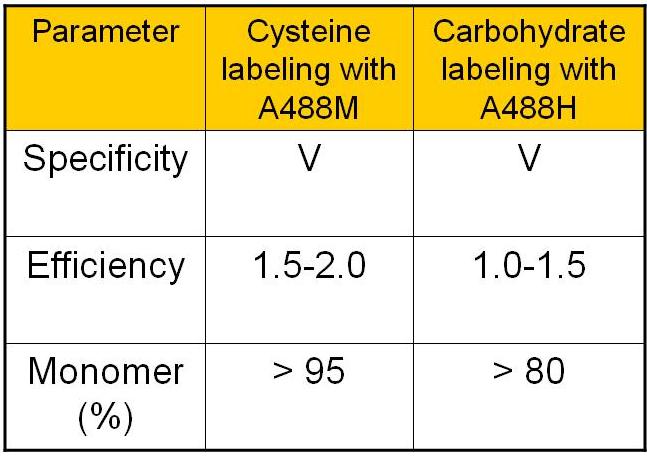
**

**G**

| Variant,  Fluorophores | Time 0 hrs  I_D_ / I_A_ | Time 2 hrs  I_D_ / I_A_ | Distance at 0 hrs, r_0_ (Å) | Distance at 2 hrs, r_2_ (Å) | ∆ distance  r_2_ - r_0_, (Å) |
| --- | --- | --- | --- | --- | --- |
| V1 A350H A488M | 0.27 | 0.73 | 40.2 | 47.4 | 7.2 |
| V2 A350H A488M | 0.21 | 0.85 | 38.6 | 48.6 | 10.0 |
| V3 A350H A488M | 0.45 | 0.72 | 43.7 | 47.2 | 3.5 |
| V4 A350H A488M | 0.30 | 0.60 | 40.8 | 45.8 | 5.0 |
| WT A350H  WT A488H | 1.69 | 1.11 | 54.6 | 51.0 | - 3.6 |

**Figure S2.** **Results from FRET experiments.**

(A) Protein gels for quality control of unlabeled and labeled cysteine variants 1 and 4. Fluorescence images were taken under UV-light exposure. Pictures of the same gels were taken also after staining with Coomassie Blue and destaining. Reducing SDS-PAGE differentiates between heavy chain and light chain labeling. (B) Glycosylation profile of variants 1 and 4. Percentage of the predominant glycoforms is listed. (C) Structures of the fluorophores used in this study for labeling cysteines and carbohydrates. The structures and molecular weights are form [www.invitrogen.com](http://www.invitrogen.com/). (D) Site-specific labeling of the cysteines of Variants 1-4 shown by gel electrophoresis of reduced protein samples pre-digested with Glu-C and pronase. (E) Site-specific labeling of wild type antibody carbohydrates with Alexa 488 hydrazide shown by gel electrophoresis of reduced antibody samples not treated “-“ or treated “+” with PNGase F. (F) Summary of specificity and efficiency of labeling at the engineered cysteines or at the native carbohydrates. Monomer levels are from SEC-HPLC analysis of labeled samples. (G) Calculated distance from FRET experiments.
