# Supplementary material for: Dynamic Fluctuations of Protein-Carbohydrate Interactions Promote Protein Aggregation
Source: PLoS One. 2009 Dec 23;4(12):e8425. doi: 10.1371/journal.pone.0008425 (PMC2791859; doi:10.1371/journal.pone.0008425)
Supplement: Figure S3 — Structural comparison of wild type and Variant FS (0.77 MB DOC) [file pone.0008425.s003.doc]

**Figure S3**

**A**


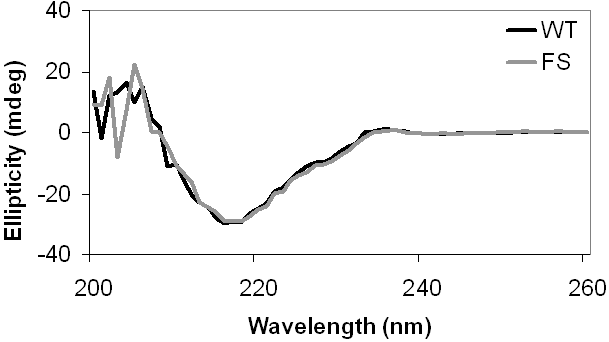


**B C D**


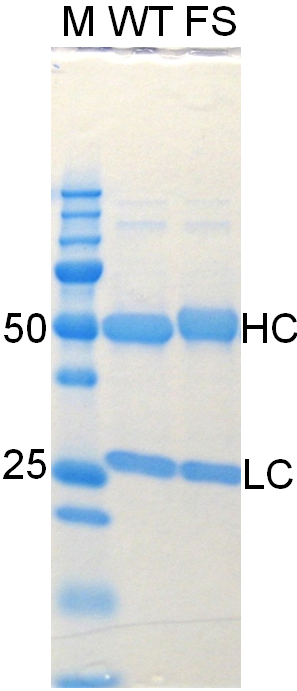

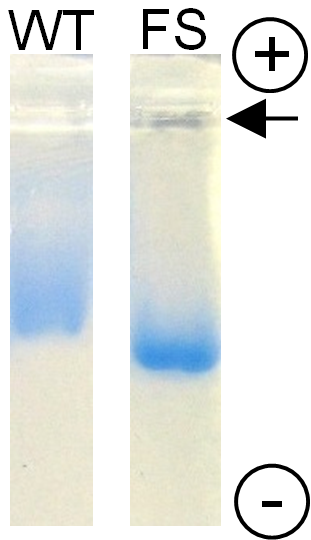

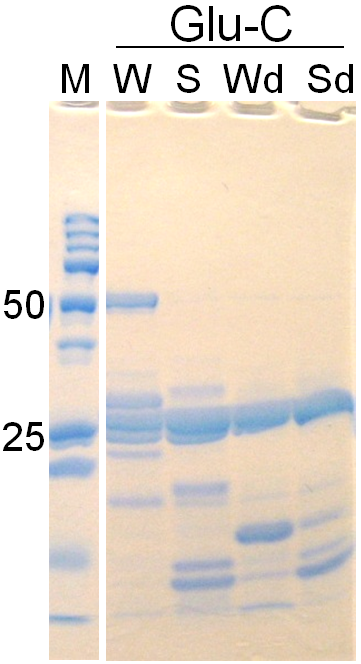


**Figure S3.** **Structural comparison of wild type and Variant FS.**

(A) CD spectra. (B) Mobility pattern on reducing SDS-PAGE. In the molecular weight ladder “M,” 50 and 25 are in kDa. (C) Mobility pattern in native gel electrophoresis. The signs “+” and “-“ refer to the anode and cathode respectively in the native gel. The arrow indicates the sample loading point. (D) Proteolytic treatment. Antibody wild type, W, and Variant FS, S, and their deglycosylated counterparts, Wd and Sd, were treated with the endopeptidase Glu-C for site-specific protein digest. The samples were resolved on 12% SDS-PAGE under reducing conditions.
